# Maintaining High Eudaimonic Wellbeing Despite Ambiguity Intolerance Among Three Employment Status Groups: Examining the Buffering Effects of Positive Psychological Attributes

**DOI:** 10.1007/s41042-021-00051-1

**Published:** 2021-03-30

**Authors:** Martin Mabunda Baluku, Edward Bantu, Betty Namale, Kathleen Otto

**Affiliations:** 1 Department of Educational, Organizational and Social Psychology, College of Humanities and Social Sciences, Makerere University, P.O Box 7062, Kampala, Uganda; 2 Work and Organizational Psychology, Faculty of Psychology, Philipps-Universität, Gutenberg Str. 18, D-35032 Marburg, Germany; 3 Faculty of Education & Human Resource, Kisii University, Kisii, Kenya

**Keywords:** Ambiguity intolerance, Basic psychological needs, Eudaimonic, Locus of control, Meaning in life, Psychological capital, Psychological wellbeing, Self-employment, Unemployment

## Abstract

The unemployed, as well as individuals in self and salaried employment, face several work-related risks and uncertainties which can result in diminished psychological wellbeing especially for individuals with high ambiguity intolerance. However, positive psychology literature suggests that individuals with strong psychological resources can be resilient in difficult circumstances. Using a sample of 922 individuals (including 240 unemployed, 391 salary-employed, and 291 self-employed) from Uganda and Kenya, we investigated the moderating effects of locus of control and psychological capital on the association between ambiguity intolerance and eudaimonic wellbeing, comparing the unemployed with individuals in salaried and self-employment. Our findings indicated that ambiguity intolerance and external locus of control are negatively associated with eudaimonic wellbeing. Conversely, internal locus of control and psychological capital were positively associated with eudaimonic wellbeing. The moderation analysis revealed that whereas an external locus of control boosts the negative effects of ambiguity intolerance on eudaimonic wellbeing, internal locus of control and psychological capital buffer against the negative effects of ambiguity intolerance on eudaimonic wellbeing. Differences between employment status groups and implications are discussed.

## Introduction

Employment is an important part of people’s lives and therefore critical to higher-order life outcomes including psychological wellbeing. Extant literature suggests that employment status and employment experiences impact psychological wellbeing (Aycan & Berry, 1996; Bonanomi & Rosina, 2020; Winefield & Tiggemann, 1990) whereby advance in employment and career foster psychological wellbeing (Welters et al., 2014; Winefield et al., 1991). The Self-Determination Theory (SDT) suggests that work that is interesting and enjoyable to an individual promotes eudaimonic living thus facilitates psychological health (Ilies et al., 2016; Ryan et al., 2013). Whereas work is expected to boost psychological growth and meaning in life, some jobs or work experiences could be detrimental to psychological wellbeing. The present study explores whether the locus of control and psychological capital are resources that can boost the eudaimonic wellbeing of individuals in different employment statuses (unemployment, self-employment, and salaried employment).

Self-employment (including own-account workers) involves several risks and uncertainties that can potentially harm the psychological wellbeing of an individual, yet some self-employed tend to report high levels of psychological wellbeing (Baron et al., 2016). Similarly, recent studies have highlighted the psychological distress of unemployment (Bartelink et al., 2020; Bonanomi & Rosina, 2020; Farré et al., 2018). However, questions have been asked whether any job is better than no job that is concerning the effects of unemployment and poor quality jobs on psychological health? (Grün et al., 2010; Otto & Dalbert, 2013). Whereas the three employment statuses are qualitatively and quantitatively different, they all tend to be characterized by high levels of uncertainty. This could lead to lowered psychological wellbeing especially for individuals who have a low tolerance for ambiguity. The current study examines whether the locus of control and psychological wellbeing can be resources that buffer against the negative effects of ambiguity intolerance on psychological wellbeing across the three employment status groups?

The recent economic crisis and the present Corona Virus Disease (COVID-19) pandemic-related economic lockdowns have had worrying effects on jobs and working lives (Doran & Fingleton, 2016; Spurk & Straub, 2020). Such situations in addition to the surge in youth populations in some parts of the world have facilitated an increase not only in the number of people in unemployment (Ackah-Baidoo, 2016; Awad, 2019; Bruhn, 2020; Liotti, 2020; Wilkinson et al., 2017) but also those in insecure and precarious employment (Gutiérrez-Barbarrusa, 2016; Kottwitz et al., 2017; Ståhl & MacEachen, 2020).

In line with previous research, we argue that unemployment is bad for psychological wellbeing. From the self-determination theory (Deci & Ryan, 1980, 2011; Ryan, 2009), it can be argued that work or being in some occupation provides a platform for fulfilling basic psychological needs thus contributing directly to psychological wellbeing. Beyond the loss of income, the deprivation theory suggests that being without work deprives individuals of several things such as time structure of the day, status, and identity, regular interactions with peers and people with similar goals (see Jahoda, 1988; Stavrova et al., 2011) which may heighten ambiguity in the lives of the unemployed. Besides, when people become unemployed, they are likely to be uncertain of when and where they will return to work, which kind of work will be available to them, whether they will be competitive enough again in the labor market, taking care of personal and family survival needs, among others. These tend to have implications on how people feel when they get re-employed. Individuals may not feel better and sometimes feel worse after prolonged periods of unemployment (Otto, Baluku, Fasbender, et al., 2020).

Whereas extant literature suggests that it is unemployment that is most detrimental to psychological wellbeing, some forms of work can make people feel worse than being unemployed. In circumstances where most work is described as precarious, individuals in salaried employment and unemployment face similar challenges including threats to work-related social needs and identities (Angrave & Charlwood, 2015; Selenko et al., 2018). The majority of jobs in less developed countries, at least in the region of Sub-Saharan Africa, are considered precarious (Desmond & Gershenson, 2016; Feder & Yu, 2019). Precarious employment includes irregular, unstable, non-standard, temporary, part-time, and short contract employment and underemployment (Kim et al., 2008). These put job holders at a disadvantage in terms of pay and social protections. Moreover, the self-employed may not be any better. Although research has generally indicated that the self-employed tend to be happier, many individuals are engaged in what has been called precarious self-employment (Kottwitz et al., 2017; Schummer et al., 2019) especially those doing business in the informal sector and the solo self-employed. These are challenged by long working hours, the burden of bearing risks, and having little social protection that affects the psychological wellbeing of entrepreneurs and the self-employed (Baron et al., 2016; Otto, Baluku, Hünefeld, et al., 2020). Overall precarious work, whether in self-employment or salaried employment is associated with insecurity, job strain, poor health, and poor psychological resources for work (Ek et al., 2014; Sharaf & Rashad, 2020). In the present paper, we propose that the extent to which individuals tolerate such uncertainties is directly linked to their level of psychological wellbeing.

There has been a surge in research on mental health challenges associated with different employment statuses, particularly in the aftermath of the economic crisis given the terrible effects it had on the labor market. However, there is limited focus on psychological resources individuals in these employment statuses use to achieve or maintain high psychological wellbeing. In line with the SDT (Deci & Ryan, 1980, 2011), perceived locus of causality could be associated with the amount of anxiety or fear following the loss of a job. Regarding self-employment, locus of control is one of the constructs that have been used to explain the so-called entrepreneurial personality (e.g., Brandstätter, 2011; Hansemark, 2003). Generally, locus of control is linked to the basic psychological needs, such that externality of control tends to thwart while internality of control tends to support these needs (Deci & Ryan, 2011). Our focus is not on the goal contents as is often the focus in SDT research, rather on how well individuals in the different employment statuses feel they are in control of their affairs and their impact on wellbeing. Additionally, we suggest that particularly psychological capital is another useful psychological resource for reducing the impact of intolerance to the uncertainty involved in different employment statuses. Psychological capital is considered a state of psychological development involving self-efficacy, optimism, hope, and resilience (Luthans, Avolio, and Avey 2007; Luthans and Youssef 2007). These resources could be useful in enabling individuals to remain resilient or cope with the realities of their work situations, hence essential in maintaining vitality and psychological wellbeing (Baumann & Eiroa-Orosa, 2016; Luthans et al., 2004).

In the present study, we argue that intolerance to ambiguity may increase the likelihood of low psychological wellbeing of individuals in different employment statuses. We further demonstrate that particularly high internal locus of control and psychological capital are important resources that buffer against the negative effects of ambiguity intolerance on psychological wellbeing. By completing the present study, we contribute to the discourse of psychological resources that are essential for the psychological wellbeing of individuals that are in precarious career situations. The remainder of the paper is structured as follows. We begin with the theoretical review and development of hypotheses and then proceed to the presentation of the methods and results and conclude with the discussion of our findings and their theoretical and practical implications.

## Theoretical Framework and Hypotheses

Research on wellbeing is mainly based on the hedonia and eudaimonia traditions. Whereas the hedonic tradition emphasizes pleasure and pain avoidance, eudaimonia focuses on living well including human flourishing and prosperity. Accordingly, eudaimonia is seen as offering a more complete understanding of psychological wellbeing (Heintzelman, 2018) given that human flourishing is a higher-order outcome than pleasure maximization and pain avoidance (Heintzelman, 2018; Samman, 2007). The present study is based on the Self-Determination Theory (SDT) given its strong emphasis on living well (Ryan et al., 2008, 2013). SDT underscores human flourishing which includes the satisfaction of three basic psychological needs: the need for autonomy, need for competence, and need for relatedness (Ryan, 2009; Ryan et al., 2008, 2013) and meaning in life (Di Fabio & Palazzeschi, 2015; Samman, 2007). This also aligns with Ryff’s model of psychological wellbeing (Ryff & Keyes, 1995; Ryff, 1995a and b, 2019; Ryff & Singer, 2008). The need for autonomy aligns with Ryff’s dimension of autonomy. The need for competence reflects environmental mastery and personal growth in Ryff’s model. The need for relatedness aligns with Ryff’s dimension of positive relations with others, and finally, meaning in life reflects the purpose in life and self-acceptance aspects of Ryff’s model. The Basic Personal Needs Theory (a micro theory within the SDT) highlights that psychological needs are important aspects of wellness. Besides these convergences, it has been claimed that personal growth (represented by the satisfaction of basic psychological needs) and meaning in life are the most eudaimonic facets of wellbeing (Ryff & Singer, 2008; Samman, 2007). Hence, our measurement of eudaimonic wellbeing focuses on these facets.

**Fig. 1:**
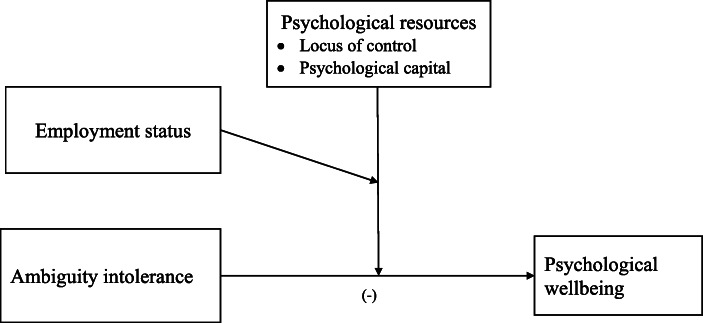
Conceptual model

The SDT postulates that eudaimonia is a way of living in which individuals predominantly focus on what is intrinsically valued, which are essential for the satisfaction of the basic psychological needs (Ryan et al., 2008). This way of living is grounded in several conceptual orientations including personality processes, personal development, individuation, mental health, self-actualization, and human functioning (Ryff & Singer, 2008) which indicate that individual differences are essential in explaining psychological wellbeing. Extant research indicates that individual differences play an important role in understanding variations in levels of psychological wellbeing (e.g., Adler & Fagley, 2005; Martin, Puhlik-Doris, Larsen, Gray, & Weir, 2003; Ryff, 1995a and b). In the present study, we focus on the role of ambiguity intolerance and psychological strengths. We propose that locus of control and psychological capital are psychological resources that enable individuals in different employment statuses to maintain high levels of psychological wellbeing. Such psychological strengths are important to drivers of psychological wellbeing (Lomas, 2019; Munoz et al., 2016). Psychological capital and particularly internal locus of control are constructs within positive psychology that reflect individual differences in response to events and contexts, therefore important for eudaimonic wellbeing. Such positive constructs enable the understanding of how people thrive, as opposed to the predominant research in psychology that focuses on alleviation of suffering and dysfunction (Adler & Fagley, 2005). To illustrate the usefulness of locality of control in wellbeing, Diener, Lucas, & Scollon (2009) observe that individuals differ in their adaptation to circumstances and events. Similarly, psychological capital is a trait-like and state-like construct (Luthans and Youssef-Morgan 2017; Luthans and Youssef 2007) that is associated with adaptability and coping with adverse conditions (Baron et al., 2016; Hicks & Knies, 2015). This forms the basis for our investigation of locus of control and psychological capital as possible moderators of the association between uncertainty tolerance and eudaimonic wellbeing among individuals in the three employment status groups. Our conceptual framework is presented in Fig. 1.

### Employment Status, Ambiguity, and Psychological Wellbeing

Based on the assumption that personal growth and meaning in life are the most eudaimonic facets of wellbeing (Ryff & Singer, 2008; Samman, 2007), the present study measures eudaimonic wellbeing with the satisfaction of Basic Psychological Needs (BPNs) comprising of autonomy, competence, and relatedness in addition to meaning in life. The satisfaction of these needs represents psychological growth. Although the SDT argues that autonomy, competence, and relatedness boost wellbeing, other theoretical orientations include these concepts in the description of psychological wellbeing (Ryff & Keyes, 1995; Ryff & Singer, 2008; Samman, 2007). The SDT theory posits that BPNs can be supported or thwarted by the context, thus impacting on wellbeing (Deci & Ryan, 1980, 2008; Deci & Ryan, 2011). The satisfaction of these needs during unemployment is critical for psychological wellbeing (Chen et al., 2015). This also applies to salaried and self-employment. However, these employment statuses involve several uncertainties which in turn are a precedent for lowered psychological wellbeing.

Available evidence suggests that intolerance to ambiguities and uncertainties affects psychological wellbeing (Çevik & Yağmur, 2018). In the current study, we argue that intolerance to the ambiguities involved in unemployment, salaried, and self-employment is the likely cause for reduced eudaimonic wellbeing. Specifically, in precarious employment situations, people worry about the risk of job loss, or the risk of never finding a job again, or the risk of business failure. These are associated with further worries such as poverty and family disruptions. We, therefore, contend that individuals who have high ambiguity intolerance are likely to experience lower psychological wellbeing than those with a high tolerance for ambiguity. Ambiguity or uncertainty tolerance is a concept within the national cultural dimensions (Hofstede et al., 2010; Hofstede & Bond, 1984). Whereas the link between culture and psychological wellbeing has not received adequate attention in scientific research, Christopher (1999) proposes that the understanding of the concept of psychological wellbeing, its theories, and measures represent an aggregation of cultural values and assumptions. That is, understanding psychological wellbeing requires an understanding of moral goals that are ingrained in a given culture. Accordingly, the conceptions of eudaimonia are culturally influenced. In this direction, SDT is considered a macro theory of several constructs including wellbeing and the social-cultural factors that determine and sustain it (Ryan, 2009). Ambiguity intolerance implies that an individual feels threatened by ambiguous situations (Yoo et al., 2011).

High uncertainty intolerance is correlated to the perception of constraints (Buhr & Dugas, 2006); suggesting that ambiguity intolerant individuals may tend to perceive more challenges. Such perceptions can lower one’s sense of control leading to negative reactions (Hong & Lee, 2015). The perceptions of constraints also have implications for the satisfaction of BPNs, and therefore the overall psychological wellbeing. Previous research has for example showed that tolerance for ambiguity has a positive relationship with perceived mastery or competence (Buhr & Dugas, 2002), which is one of the dimensions of psychological wellbeing (Ryff & Keyes, 1995; Samman, 2007). On the contrary, ambiguity intolerance is correlated to worry (Buhr & Dugas, 2006) and depression (Enoki et al., 2019), hence could negatively predict eudaimonic wellbeing. Also, high intolerance for uncertainty is related to poor psychological adjustment (Kurita et al., 2013) and coping (Mitmansgruber et al., 2016), an indication of thwarted psychological growth. Further empirical evidence shows that ambiguous situations and intolerance for uncertainty are related to mental health challenges including disorders such as anxiety and social withdrawal (Fergus, 2013; Zimmer-Gembeck & Nesdale, 2013). Based on this empirical evidence, we hypothesize that ambiguity intolerance predicts the low levels of eudaimonic wellbeing.

Hypothesis 1. Ambiguity intolerance is negatively associated with eudaimonic wellbeing.

Although individuals in different employment statuses experience uncertainties in their daily lives, the nature of uncertainties they face vary. We particularly presuppose that the unemployed have lower psychological wellbeing than individuals in salaried and self-employment. Extant literature on the effects of employment status shows a strong correlation between unemployment and low wellbeing measured in terms of happiness or life satisfaction (Knabe et al., 2010; van der Meer, 2014). Often, the unemployed report lowered happiness and satisfaction than the employed (Stavrova et al., 2011; Winkelmann & Winkelmann, 1998). This is associated with several things that change in life leading to mental health challenges among the unemployed (Elovainio et al., 2001; Frese & Mohr, 1987). At the extreme, the risks of mortality cannot be ruled out (Otto, Baluku, Fasbender, et al., 2020). In this direction, an earlier study by Grobe (2006) revealed that the risk to die is 3.9 times higher for the long-term unemployed than for those continuously employed.

In social-cultural terms, unemployment is considered a deviation from the norm in societies that value work (Carroll, 2007; Helliwell & Huang, 2014). It has also been found to negatively impact people’s ability to engage in leisure and social activities, the formation of interpersonal relationships, childbearing, and family development (Choudhury, 2017; Kunze & Suppa, 2017). In this direction, it has been posited that whereas being unemployed may relate to low psychological wellbeing, it is the social norm that may explain the differences in psychological wellbeing among the unemployed and other members of society (Helliwell & Huang, 2014).

In economic terms, unemployment leads to loss of income, moreover beyond salary, including losses relating to skills development (Carroll, 2007). These losses may result in uncertainty about the survival costs and possibilities of future employment. Whereas unemployment benefits (unemployment insurance or unemployment compensation) can mitigate against these economic uncertainties (Sjoberg, 2010), the situation can become depressing in places where unemployment benefits are non-existent. In addition, there are worries about possibilities never returning to work, given that they sometimes face discrimination in the labor market arising from the preference for hiring already working individuals (Kugler & Saint-Paul, 2004). These uncertainties may facilitate what Knabe et al. (2010) labeled as the “saddening effect,” denoting that the unemployed are often less contended, more stressed, and sad than their employed counterparts when in a similar event or activity. The conditions in the labor market however could be a contributory factor. Previous findings have revealed that unemployment results in higher distress when or where unemployment rates are high (Chadi, 2014) suggesting that being unemployed in the current context of a growing unemployment crisis is related to higher distress and lowered psychological wellbeing.

However, the unemployed may not be worse than some in salaried and self-employment who are in bad jobs. This study refers to young people in East Africa, which is a region where most jobs are considered bad, precarious, while the majority of the working individuals are considered working poor (Desmond & Gershenson, 2016; Siegmann & Schiphorst, 2016). Extant research evidence shows that those in such working conditions have poor wellbeing just like those in non-employment (Otto & Dalbert, 2013; Vancea & Utzet, 2017). Like unemployment, being in less interesting work thwarts satisfaction of basic psychological needs (Kim & Allan, 2019) and may affect social relations (Vancea & Utzet, 2017) and career identity. This is in addition to worries over high layoff rates, inadequate income, and lack of opportunity to optimize one’s competencies. Nonetheless, the certainty of some income, ability to structure time, relating with people in the workplace, and other benefits of work should provide a basis for better psychological wellbeing than being unemployed. Among the different employment status groups, the self-employed are expected to exhibit higher levels of psychological wellbeing. Even when their incomes are low, the self-employed are happier and satisfied (Berglund et al., 2015; Schneck, 2014) because of high levels of independence experiences in this form of employment (Schneck, 2014). Considering the review in this subsection, we hypothesize that:

Hypothesis 2. The unemployed have lower eudaimonic wellbeing than their counterparts in salaried and self-employment.

### Positive Psychological Attributes as Coping Resources

If ambiguity intolerance is negatively related to psychological wellbeing, can this association be weakened or boosted by one’s locality of control and level of psychological capital? And can this be relevant among individuals in unemployment, salaried, and self-employment? We particularly posit that an internal locus of control and high positive psychological capital are resources individuals can use to maintain high psychological wellbeing in all three employment statuses. The self-determination theory particularly emphasizes the role of perceived causality and perceived competence (Ryan & Deci, 2002) in maintaining wellness. The perceived causality points to the role of locus of control, while competence perceptions relate to the efficacy perceptions thus highlighting the role of psychological capital. Both positive psychological capital and particularly internal locus of control emphasize individuals’ strengths (Ajzen 2002; Luthans et al., 2004) and therefore are useful in dealing with ambiguities in life and work (Buddelmeyer & Powdthavee, 2016; Kormanik & Rocco, 2009).

Locus of control in this regard concerns the extent to which the external environment is perceived as facilitating or hindering personal growth. Rotter (1966) conjectured locus of control as the predisposition in the expectation of internal or external control of reinforcement. An internal locus of control is the predisposition of expectation that outcomes are largely dependent on one’s behavior or traits while an external locus of control refers to the predisposition to expect that outcomes are determined by some factors beyond one’s control (Levenson, 1973; Rotter, 1966). Such external factors may include chance, powerful others, luck, and fate (Kormanik & Rocco, 2009; Levenson, 1973). Consequently, internal locus of control is viewed in positive terms and associated with positive outcomes (Galvin et al., 2018) such as embracing positive values, exhibiting a high level of confidence, self-esteem, intrinsic motivation, job satisfaction, and coping with uncertainty and challenges (Buddelmeyer & Powdthavee, 2016; Cvetanovski & Jex, 1994; Luthans, 2006; Ng et al., 2006). However, (Galvin et al., 2018) point out that external locus of control is also useful in certain situations, for example, their lower propensity for lower self-blame enables them to cope with pressure.

The assumptions of the SDT provide us with an understanding of how locus of control affects motivation and gratification of basic psychological needs. Particularly, it is suggested that the need for autonomy is affected by the locality of control (Ryan & Deci, 2002; Ryan & Deci, 2008). Beyond the basic psychological needs, internal locus of control is generally positively associated with wellbeing both in work and non-work situations (Galvin et al., 2018; Ng et al., 2006; Spector et al., 2002), including during unemployment (Cvetanovski & Jex, 1994). It is reported that internal locus of control is associated with lower levels of anxiety and depressive symptoms during unemployment and times of financial hardships (Cvetanovski & Jex, 1994; Frankham et al., 2019). However, there are aspects of external locus of control that enable individuals high on this attribute to maintain high-level wellbeing during uncertainty, especially their tendency not to blame themselves for the bad outcomes (Galvin et al., 2018). However, when external factors are blamed for being unemployed or having a bad job, it can increase the negative feeling about the job or lower the optimism for obtaining employment.

We, therefore, posit that individuals with an external locus of control are likely to experience low eudaimonic wellbeing, compared to those with an internal locus of control. This further derives from the assumption that the context presents a controlling effect, particularly the pressure it exerts on the individual leads individuals to external attributions of causality (Ryan & Deci, 2002), which undermines coping with uncertainties relating to unemployment, consequently impacting negatively on growth and wellness. However, this may depend on whether unemployment is involuntary or voluntary. In the case of involuntary unemployment, we expect that individuals would adopt a more external locus of control. On the other hand, internal locus of control is likely to boost coping; thus, individuals are likely to maintain high psychological wellbeing during unemployment. Moreover, this relationship may be expected among individuals of different employment statuses, given that the effects of locus of control tend to be universal (Spector et al., 2002). Overall, we hypothesize that:

Hypothesis 3a. Locus of control has significant effects on eudaimonic wellbeing.

Hypothesis 3b. Locus of control moderates the association between ambiguity intolerance and eudaimonic wellbeing, such that the effects of ambiguity intolerance are low for individuals higher on internal locus of control across the three employment status groups.

Psychological capital is the other psychological resource we are studying as being important to maintaining a high level of psychological wellbeing when individuals are unemployed or facing uncertainties in their jobs. The psychological capital theory highlights four resources including confidence (efficacy), hope, resilience, and optimism which constitute what is termed positive psychological capital (Luthans, Avolio, Avey, & Norman, 2007; Luthans et al., 2004; Luthans & Youssef-Morgan, 2017). The theory emphasizes strengths, health, and vitality over weaknesses, disease, and pathology (Luthans et al., 2004). In this regard, it has been noted that psychological capital should be positively related to psychological wellbeing (Avey et al., 2010; Baluku et al., 2018).

Applied to business, work, and unemployment contexts, we expect psychological capital to buffer against the negative impact of ambiguity intolerance on the eudaimonic wellbeing of individuals in the three employment statuses. Towards this direction, previous research has established that psychological capital is a critical response in managing and preventing stress in work and business situations (Avey et al., 2009; Baron et al., 2016). Currently, there is limited literature on the relationship between psychological capital and eudaimonic wellbeing in different populations. However, a study of this relationship among employees revealed that psychological capital was positively related to both the index of psychological wellbeing and general health measures (Avey et al., 2010). Cole, Daly, and Mak (2009) from their economic analysis report findings support this assumption. They show that psychological capital has an influence on the effects of being unemployed on psychological wellbeing and increases chances of re-employment, while low psychological capital is associated with the higher risk of being unemployed. For those in self-employment, psychological capital is one of the reasons why some individuals in self-employment maintain high psychological wellbeing and have low levels of stress despite the numerous challenges involved in their daily work roles (Baron et al., 2016). Psychological capital is important not only to those in unemployment or owning businesses but also to those in precarious life circumstances including difficult work circumstances (Bak-Klimek et al., 2014).

Although psychological resources are essential for adjustment and coping to uncertainty and difficult life circumstances, its role in buffering against the effects of ambiguity intolerance on wellbeing might be affected by the prevailing economic and socio-cultural conditions. Economic research on the effect of unemployment on wellbeing indicates the impact of factors such as level of unemployment, the social norm to work, and social security systems (Carroll, 2007; Helliwell & Huang, 2014). This may not only affect the amount of psychological capital unemployed individuals possess given that context tends to not just influence the amount of psychological capital (Baur et al., 2018), but also how they apply it in coping with the uncertainties they face as a result of being unemployed or having a precarious job or the uncertainties of doing business. Accordingly, we expected the unemployed to report lower levels of psychological capital than their counterparts in salaried and self-employment. However, unemployed individuals with higher psychological capital are also likely to report higher levels of eudaimonic wellbeing.

Hypothesis 4a. The unemployed have lower levels of psychological capital than the salary- and self-employed individuals.

Hypothesis 4b. Psychological capital is positively associated with high eudaimonic wellbeing.

Hypothesis 4c. Psychological capital moderates the impact of ambiguity intolerance on eudaimonic wellbeing such that the impact of ambiguity intolerance is low when psychological capital is high across the three employment statuses.

## Methods

### The Sample and Procedure

The study was conducted among unemployed, salary-employed, and self-employed individuals from Uganda and Kenya. In Uganda, the study was carried out in the Capital City, Kampala, while in Kenya, the study was conducted in two cities of Kisii and Maseno. Overall, the sample comprised 922 participants. The unemployed sampled totaled 240 individuals; 184 Ugandans (54.35% males) and 56 Kenyans (58.93% males). The salary-employed sample comprised of 391 participants including 242 Ugandans (54.13% males) and 149 Kenyans (51.01% males). Lastly, the self-employed sample comprised 291 participants including 167 Ugandans (55.09% males) and 124 Kenyans (57.56% females). Participants were recruited using different approaches depending on their employment status. The unemployed sample was recruited through various social forums, associations, and events for the youth. The self-employed sample was recruited through entrepreneurs’ forums including meetings and workshops. Some were approached at their business premises and requested to participate in the study. Finally, the salary-employed sample was recruited from various organizations within the specified cities with the support of the organizations’ human resources offices. The study targeted young unemployed, salary-employed, and self-employed individuals in the age range of 18 to 35 years (average age = 25.08 years, SD = 3.50). The majority of the participants were highly educated, with 51.4% having obtained a bachelor’s degree or higher and 35.3% had completed ordinary or advanced technical or vocational courses.

### Measures

#### Ambiguity Intolerance

Ambiguity intolerance was measured using the Personal Cultural Orientation scale (Sharma, 2010). The scale operationalizes Hofstede’s dimensions (Hofstede et al., 2010) at the personal level. The scale measures the ambiguity intolerance dimension with four (4) items. These were assessed on a 7-point Likert scale (1 = strongly disagree to 7 = strongly agree). A sample item is “I feel stressed when I cannot predict consequences.” These items showed an acceptable level of internal consistency with *α* = .79 for the present study.

#### Locus of Control

Locus of control was measured using the Multidimensional LOC scale (Levenson, 1973). This scale comprises 24 items measuring internal and external locus of control. The scale further focuses on the sub-dimensions of external locus of control (powerful others and chance). In this paper, however, we focus on the two major dimensions of internal and external locus of control. Sample items include “When I make plans, I am almost certain to make them work” (internal locus of control) and “My life is chiefly controlled by powerful others” (external locus of control). The items were measured on a 6-point Likert scale (1 = strongly disagree, 6 = strongly agree) and showed acceptable internal consistencies (*α* = .71 and .79 for internal and external dimensions respectively for the current study), above the threshold of .70 (Nunnally, 1978).

#### Psychological Capital

Psychological capital was measured using the Psychological Capital Questionnaire (PCQ24: Luthans, Avolio, & Avey, 2007). The questionnaire consists of 24 items measuring four psychological resources (confidence/ self-efficacy, hope, resilience, and optimism) that constitute the construct psychological capital (Luthans, Avolio, & Avey, 2007; Luthans et al., 2004; Luthans & Youssef-Morgan, 2017). Items are measured on a 6-point Likert scale requiring participants to indicate their level of agreement with the 24 statements (1 for strongly disagree and 6 for strongly agree). A sample item is “I feel confident contacting other people to discuss problems.” For the current study, the questionnaire had a high reliability coefficient (α = .88).

#### Psychological Wellbeing

Samman (2007) proposes a questionnaire for psychological wellbeing that contains items from the Deci and Ryan Basic Psychological Needs short scale and the Meaning in Life questionnaire (Steger et al., 2006). The questionnaire comprises 12 items, three items reflecting each dimension. All items are measured on a 4-point scale from 1 (not at all true) to 4 (completely true). Sample items on each aspect read: “I feel like I can pretty much be myself in daily situations” (autonomy); “most days I feel a sense of accomplishment from what I do” (competence); “People in my life care about me” (relatedness); and “My life has a clear sense of purpose” (meaning in life). For the current study, the questionnaire showed a strong internal consistency (*α* = .91).

### Analytical Strategy

To test our hypotheses, we used the PROCESS macro v3.4 (Hayes, 2017). Given that individuals’ demographic characteristics tend to influence psychological wellbeing (Arafa et al., 2003; Shin et al., 2007), we included age, sex, country, and level of education as control variables in our analyses. As reflected in the conceptual model (Fig. 1), the study examines the moderation effects of locus of control and psychological capital in the relationship between ambiguity intolerance and psychological wellbeing for the three forms of employment status (unemployed, salary-employed, and self-employed). Hence, a moderated moderation analysis is applied using Model 3 of the PROCESS Macro v3.4 (Hayes, 2017). This model computes the double moderation effects simultaneously. Three regression models were computed. The first model explored the moderation effects of internal locus of control (i.e., interactive effects of ambiguity intolerance, locus of control, and employment status); the second test the moderation effects of external locus of control; and the third focuses on the moderation effects of psychological capital. All variables defining the product terms for the moderations were centered. Employment status as a multi-categorical variable—comprising the three categories: unemployed, self-employed, and salary-employed—was automatically dummy coded by the PROCESS Macro. Unemployment status was used as the comparison indicator. Therefore, the effects of employment status are separated into two. First are the effects of self-employment (*Z*_1_) and second are the effects of salaried employment (*Z*_2_). These effects are in comparison to the effects of unemployment; thus, the positive or negative effects are interpreted as in comparison with the effects of unemployment. For the analyses, we applied sample bootstrapping at 5000 as recommended by Hayes (2013).

## Results

### Preliminary Analyses

Table 1 presents the descriptive statistics and correlations between the study variables. The correlations presented in Table 1 refer to the combined sample. The correlations between the variables were as expected. Ambiguity intolerance and external locus of control were negatively correlated to eudaimonic wellbeing. In contrast, psychological capital and internal locus of control were positively correlated to eudaimonic wellbeing. Table 2 shows the differences among employment status on ambiguity intolerance, psychological capital, and eudaimonic wellbeing. Contrary to our assumptions (hypotheses 2 and 4a), the unemployed reported higher psychological wellbeing (*M* = 2.99, SD = 1.00) than individuals in salaried employment (*M* = 2.79, SD = .84). However, those in self-employment generally reported the highest levels of psychological wellbeing (*M* = 3.10, SD = .90). Significant differences were also observed in psychological capital and internal locus of control, as can be seen in Table 2. Considering the Partial Eta Squared (*η*_*p*_^*2*^) threshold values (Cohen, 1988), the effects of employment status on eudaimonic wellbeing, psychological capital, and internal locus of control were rather small, while the effects were insubstantial for external locus of control. However, the effects on ambiguity intolerance were average (medium), with salary-employed showing higher ambiguity intolerance than the self-employed and the unemployed.

**Table 1:** Correlations and descriptive statistics

	*M* (min., max.)	SD	1	2	3	4	5
1. Ambiguity intolerance	3.24 (1, 7)	1.43	**.79**				
2. External LOC	3.16 (1, 6)	.82	.10^**^	**.71**			
3. Internal LOC	3.99 (1, 6)	.85	−.23^***^	−.16^***^	**.79**		
4. Psychological capital	4.09 (1, 6)	.84	−.25^***^	−.25^***^	.53^***^	**.88**	
5. Psychological well-being	2.94 (1, 4)	.91	−.36^***^	−.21^***^	.38^***^	.51^***^	**.91**

^**^p < .01, ^***^p < .001, *LOC* locus of control

Cronbach’s *α* in bold diagonal

**Table 3:** Regression results for moderated moderation effects on eudaimonic wellbeing

Moderator		Model 1 Internal locus of control (M1)			Model 2 External locus of control (M2)			Model 3 Psychological capital (M3)		
Predictor		*b*	SE	*t*	*b*	SE	*t*	*b*	SE	*t*
Sex		.03	.05	.48	.03	.06	.55	−.01	.05	−.12
Age		−.02	.04	−.42	.03	.04	.78	−.01	.03	−.31
Educational level		.01	.02	.37	.03	.02	1.16	.01	.02	.49
Country		−.27^***^	.05	−4.96	−.35^***^	.06	−6.10	−.30^***^	.05	−5.90
Self-employment (*Z*_1_)		−.20^*^	.08	−2.57	.04	.08	.45	−.13	.07	−1.91
Salaried-employment (*Z*_2_)		−.10	.07	−1.51	−.06	.07	−.79	−.03	.06	−.42
Ambiguity tolerance		−.15^***^	.04	−3.89	−.17^***^	.04	−4.15	−.06	.04	−1.79
Internal locus of control (M1)		.41^***^	.06	7.09						
External locus of control (M2)					−.19^**^	.07	−2.71			
Psychological capital (M3)								.77^***^	.07	11.30
Ambiguity tolerance × *M*		.15^**^	.05	3.05	−.11^*^	.05	−2.32	−.01	.04	−.17
Ambiguity tolerance × self-employment		−.13^**^	.05	−2.64	−.02	.05	−.43	−.23^***^	.05	−4.93
Ambiguity tolerance × salaried-employment		−.07	.05	−1.31	−.12^*^	.05	−2.24	−.13^**^	.05	−2.61
*M* × self-employment		.22	.12	1.81	−.25^*^	.11	−2.31	−.38^***^	.10	−3.98
*M* × salaried-employment		−.20^*^	.08	−2.55	.14	.09	1.61	−.48^***^	.09	−5.66
Ambiguity tolerance × *M* × self-employment		.20^**^	.08	2.66	−.12	.07	−1.82	−.20^***^	.05	−3.99
Ambiguity tolerance × *M* × salaried-employment		−.09	.06	−1.49	.03	.06	.51	.09	.05	1.93
Model summary		*R*^2^ = .30, *F*(15, 902) = 25.47^***^			*R*^2^ = .23, *F*(15, 902) = 18.11^***^			*R*^2^ = .41, *F*(15, 902) = 41.75^***^		
∆*R*^2^ as a result of 3-way interaction		∆*R*^2^ = .02, *F*(2, 902) = 9.18^***^			∆*R*^2^ = .01, *F*(2, 902) = 3.41^*^			∆*R*^2^ = .01, *F*(2, 902) = 8.02^***^		
Test of ambiguity intolerance × *M* interaction		Effect		*F*	Effect		*F*	Effect	SE	*F*
Unemployed		.15^**^		9.31	−.11^*^		5.36	−.01		.03
Self-employed		.35^***^		34.10	−.23^***^		23.15	.19^***^		31.19
Salary-employed		.06		3.42	−.09^*^		5.53	.08^**^		8.06
Conditional effects of ambiguity intolerance	Levels of *M*	Effect	SE	*t*	Effect	SE	*t*	Effect	SE	*t*
Unemployed	Mean − 1	−.28^***^	.06	−4.46	−.08	.06	−1.30	−.06	.05	−1.22
	Mean	−.15^***^	.04	−3.89	−.17^***^	.04	−4.15	−.06	.04	−1.79
	Mean + 1	−.03	.05	−.56	−.26^***^	.05	−5.30	−.07	.05	−1.51
Self-employed	Mean − 1	−.58^***^	.07	−8.53	−.01	.08	−.08	−.46^***^	.05	−9.24
	Mean	−.29^***^	.03	−8.90	−.19^***^	.03	−6.01	−.29^***^	.03	−9.72
	Mean + 1	.01	.05	.20	−.38^***^	.05	−7.61	−.13^***^	.03	−4.02
Salaried employment	Mean − 1	−.27^***^	.04	−6.39	−.22^***^	.04	−5.22	−.26^***^	.04	−6.53
	Mean	−.22^***^	.04	−6.29	−.29^***^	.04	−8.36	−.19^***^	.03	−5.79
	Mean + 1	−.17^***^	.05	−3.67	−.35^***^	.05	−7.80	−.12^**^	.04	−2.81

^*^p < .05, ^**^p < .01, ^***^p < .001; sex: male = 0, female = 1; country: Uganda = 0, Kenya = 1. *M* moderator, *Z* employment status

Dummy coding of employment status: unemployment status as the comparison indicator

### Testing for Moderation Effects of Locus of Control and Psychological Capital

The moderated moderation effects are presented in Table 3. Model 1 refers to the interaction effects of ambiguity intolerance, internal locus of control, and employment status on eudaimonic wellbeing. Model 2 refers to the interaction of ambiguity intolerance, external locus of control, and employment status. The last model concerns the interaction effects of ambiguity intolerance, psychological capital, and employment status. Models 1 and 2 (in Table 3) show that ambiguity intolerance was negatively associated with psychological wellbeing, supporting hypothesis 1. However, this association was not significant in model 3. The effects of employment status indicate that salaried employment was not substantially associated with eudaimonic wellbeing in all three models. On the other hand, self-employment was negatively associated with psychological wellbeing in Model 1(*b* = −.20, *p* < .05). It should be noted that these effects are in comparison to the effects of unemployment. This corroborates findings reported earlier that the unemployed had higher eudaimonic wellbeing than individuals in salaried employment (see Table 2). Concerning the direct effects of the moderator variables, results in Table 3 show that internal locus of control (*b* = .41, *p* < .001) and psychological capital (*b* = .77, *p* < .001) were positively associated with eudaimonic wellbeing. On the contrary, external locus of control was negatively associated with eudaimonic wellbeing (*b* = −.19, *p* < .001). Hence, hypotheses 3a and 4b are supported.

**Table 2:** Multivariate analysis for differences in study variables among the employment statuses

	Employment status	*M*	SD	*F*	*p*	*η*_p_^2^
Ambiguity intolerance	Unemployed	3.52	1.33	64.46	<.001	.124
	Self-employed	2.53	1.50			
	Salary-employed	3.64	1.22			
External locus of control	Unemployed	3.17	.80	.24	.694	.001
	Self-employed	3.17	.62			
	Salary-employed	3.15	.97			
Internal locus of control	Unemployed	3.98	.91	7.60	.001	.016
	Self-employed	4.09	.47			
	Salary-employed	3.92	1.00			
Psychological capital	Unemployed	4.08	.90	13.62	<.001	.029
	Self-employed	4.25	.65			
	Salary-employed	3.97	.92			
Psychological well-being	Unemployed	2.99	1.00	11.97	<.001	.026
	Self-employed	3.10	.90			
	Salary-employed	2.79	.84			

Wilks’ Lambda for the composite (*F* = 13.73, *p* < .001, *η*_*p*_^*2*^ = .070); *η*_*p*_^*2*^ partial eta squared

Controls variables: age, sex, country, level of employment

Thresholds for effect sizes (*η*_*p*_^*2*^) according to Cohen (1988): small (.01), medium (.06), and large (.14)

Results in Table 1 further refer to the moderation effects of locus of control and psychological capital on the relationship between ambiguity intolerance and eudaimonic wellbeing for the unemployed, self-employed, and salary-employed individuals. It is again important to note that employment status was dummy coded, and unemployment was used as the comparison status. As can be seen from the results of Model 1, there were substantial two-way interaction effects of ambiguity intolerance and internal locus of control on eudaimonic wellbeing (*b* = .15, *p* = .01). Similarly, the interaction effects of ambiguity intolerance and external locus of control on eudaimonic wellbeing were significant (*b* = −.11, *p* < .05). Considering the moderation effects of employment status, our results show substantial interaction effects of ambiguity intolerance and self-employment on eudaimonic wellbeing in Model 1 (*b* = −.13, *p* < .01) and the interaction effects of internal locus of control and salaried-employment (*b* = −.20, *p* < .05). Model 2 shows substantial interaction effects of ambiguity intolerance and salaried-employment (*b* = −.12, *p* < .05) as well as interaction effects of external locus of control and self-employment (*b* = −.25, *p* < .05). Concerning the three-way moderation, significant effects on eudaimonic wellbeing were observed for the interactions among ambiguity intolerance, internal locus of control, and self-employment.

**Fig. 2:**
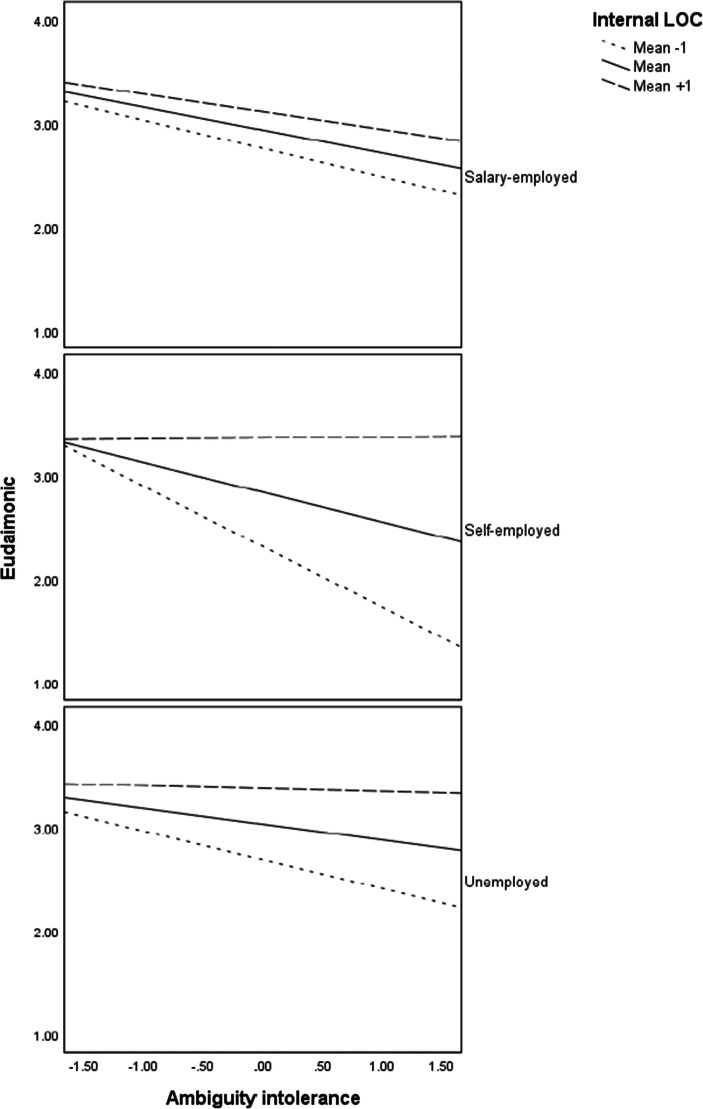
Interaction effects of ambiguity intolerance and internal locus of control on eudaimonic wellbeing (PWB) for each employment status

**Fig. 3:**
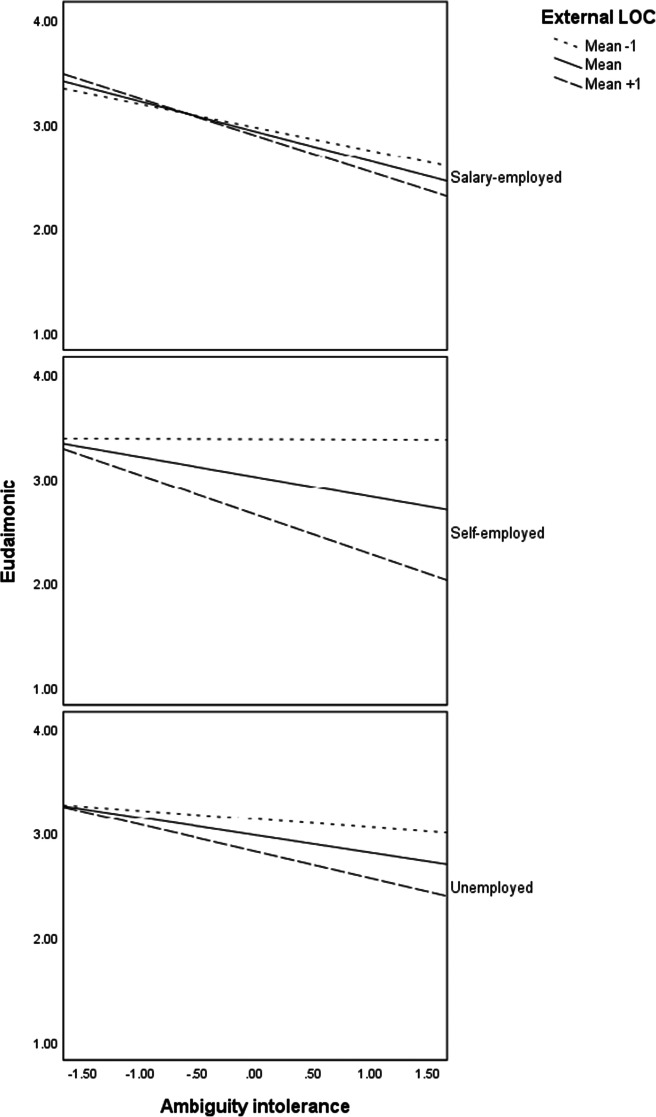
Interaction effects of ambiguity intolerance and external locus of control on eudaimonic wellbeing (PWB) for each employment status

Probing of the moderation effects reveal that there were significant interaction effects of ambiguity intolerance and internal locus of control for unemployed (*b* = .15, *p* < .01) and self-employed (*b* = .35, *p* < .001) but not for the salary-employed. On the other hand, the interaction effects of ambiguity intolerance and external locus of control were significant for all three employment statuses: unemployed (*b* = −.11, *p* < .05), self-employed (*b* = −.23, *p* < .001), and salary-employed (*b* = −.09, *p* < .05). Probing of the three-way interactions and as indicated in the regression plots in Fig. 2, individuals with a high internal locus of control (one standard deviation above the mean) maintain high levels of eudaimonic wellbeing in all the three employment status groups. Eudaimonic wellbeing tends to be relatively lower at high levels of ambiguity intolerance for individuals with a low internal locus of control (one standard deviation above the mean) in all three employment status groups. On the contrary, regression plots in Fig. 3 indicate that individuals with low external tend to maintain high eudaimonic wellbeing, especially for the self-employed and unemployed groups. Individuals with a high external locus of control tend to have low eudaimonic wellbeing especially when they also have ambiguity intolerance.

**Fig. 4:**
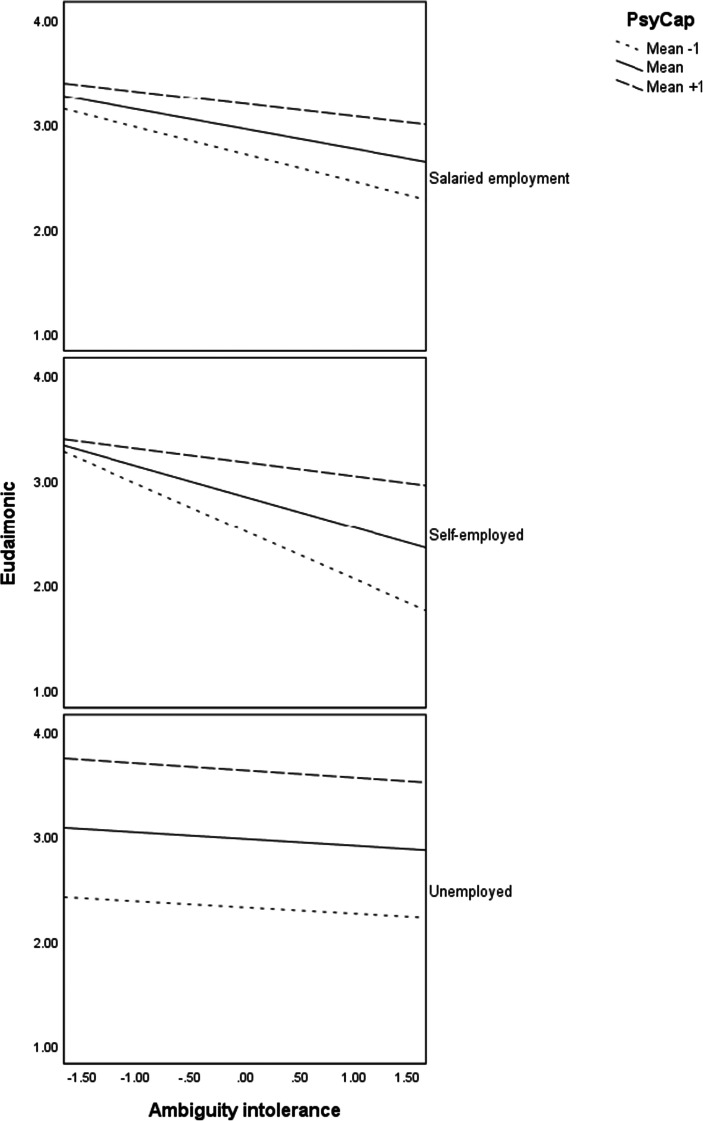
Interaction effects of ambiguity intolerance and psychological capital on eudaimonic wellbeing (PWB) for each employment status

Model 3 in Table 3 presents the moderation effects of psychological capital. Generally, the results reveal no interaction effects of ambiguity intolerance and psychological capital on eudaimonic wellbeing. Considering employment status, we found significant interaction effects of ambiguity intolerance and self-employment (*b* = −.23, *p* < .001) as well as ambiguity intolerance and salaried employment (*b* = −.13, *p* < .01) on psychological wellbeing. We also found substantial interaction effects of psychological capital and self-employment (*b* = −.38, *p* < .001) as well as psychological capital and salaried-employment (*b* = −.48, *p* < .001). Concerning the three-way interactions of ambiguity intolerance, psychological capital, and employment status, results in Table 3 show significant interaction effects for self-employment (*b* = .20, *p* < .001) but only marginal effects for salaried-employment. Probing of the interaction effects revealed that the effect of psychological capital on the relationship between ambiguity intolerance and eudaimonic wellbeing was strong for the self-employed (*b* = .19, *p* < .001) and salary-employed (*b* = .08, *p* < .01) but not for the unemployed. Probing of the three-way interactions and the regression plots in Fig. 3 indicate that although eudaimonic wellbeing for the unemployed was high at high levels of psychological capital and lower at low levels of psychological capital, the effects were quite similar at all levels of ambiguity intolerance. For the salaried and self-employed, the effects of ambiguity intolerance were higher for individuals reporting high psychological capital ambiguity intolerance (Fig. 4).

## Discussion

Positive psychological literature suggests that psychological strengths are important resources in coping with adverse and uncertain situations (Buddelmeyer & Powdthavee, 2016; Luthans & Youssef-Morgan, 2017), hence essential for attaining and maintaining vitality, happiness, and flow (Seligman, 2002; Youssef & Luthans, 2007) not only in work situations but also in life generally. However, individuals differ on these psychological resources, hence accounting for variations in psychological wellbeing (Adler & Fagley, 2005; Martin et al., 2003). We particularly examined the moderating role of locus of control and psychological capital (as psychological resources) in the association between ambiguity intolerance and eudaimonic wellbeing of individuals in different employment status groups (unemployed, self-employed, and precarious salaried-employed).

In line with previous empirical findings, our results revealed that there are substantial differences in the eudaimonic wellbeing of the unemployed, self-employed, and salary-employed. However, our hypothesis that the unemployed have the lowest levels of eudaimonic wellbeing among these three employment status groups was not supported. Whereas the unemployed reported lower eudaimonic wellbeing than the self-employed, they were better off than the salary-employed. This brings to light an important question of whether just any job is better than none (Otto, Baluku, Fasbender, et al., 2020). Whereas previous studies have indicated that the unemployed are not different from those in insecure or unsatisfactory employment and student populations in their levels of psychological wellbeing (Otto & Dalbert, 2013; Winefield et al., 1991), our results indicate that precarious jobs can be more harmful than not having a job at all. Previous studies have already highlighted the dangers of precarious or insecure jobs on mental health (Benach et al., 2016; Otto & Dalbert, 2013). Moreover, like unemployment, the psychological effects of precarious jobs accrue not only to the worker but may extend to the family as well (Benach et al., 2016). The finding that those in salaried employment have lower psychological wellbeing than the unemployed also corroborates the fact that the majority of workers in Sub-Saharan Africa fall under the so-called “working poor” (Desmond & Gershenson, 2016; Feder & Yu, 2019). Those who are extremely poor, yet having jobs, could be living in more deplorable conditions and experiencing the misery of poverty more than some of the unemployed from relatively higher socio-economic backgrounds. They work, but still, have to worry about the basic needs. In line with self-determination theory, this thwarts the realization of basic psychological needs and purpose in life.

The deprivation model suggests that unemployment deprives individuals of several things that are pertinent in life (Creed & Evans, 2002; Jahoda, 1997; Stavrova et al., 2011).

Moreover, there are further uncertainties individuals in this status are confronted with. As indicated above, some employed people are not different or even in worse situations than those who are unemployed. Moreover, the self-employed also face critical risks and uncertainties in their work (Baron et al., 2016). In support of our hypothesis (1), in all three employment statuses, ambiguity intolerance is associated with lower eudaimonic wellbeing. Given that ambiguity intolerance is associated with worry and perception of constraints (Buhr & Dugas, 2006) rather than opportunities, it follows that during unemployment or precarious employment, individuals are likely to worry more about matters such as meeting living costs, likelihoods of job loss, and ability to regain employment. In this regard, we find that those in salaried-employment and the unemployed have the highest levels of ambiguity intolerance and at the same time reported the lowest levels of eudaimonic wellbeing respectively.

The main assumption of our study (hypotheses 3 and 4) is that locus of control (specifically, internality of control) and psychological capital are resources that are useful to individuals in maintaining a high level of eudaimonic wellbeing, despite the ambiguities they face. We particularly posit that ambiguity intolerance is associated with low eudemonic wellbeing among three employment status groups. Whereas an external locus of control can boost these negative effects, we propose that internal locus of control and psychological capital buffer against the negative effects of ambiguity intolerance. Concerning the role of locus of control, first, our findings supplement extant literature showing that internal locus of control is positively associated while external locus of control is negatively associated with wellbeing (e.g., Galvin et al., 2018; Ng et al., 2006; Spector et al., 2002).

Second, we found support for the two-way interactions (hypothesis 3) between ambiguity intolerance and locus of control. Although a high level of internal locus of control buffered against the negative effects of ambiguity intolerance on eudaimonic wellbeing, high level external locus of control was found to increase the negative effects of ambiguity intolerance. Hence, the predisposition for internality of locus of control increases the likelihood of having high eudaimonic wellbeing despite when one has high intolerance for ambiguity. On the contrary, predisposition to external locality of control increases chances of low eudaimonic wellbeing especially when one’s level of intolerance for ambiguity is also high. This finding extends extant knowledge about the role of personality variables in explaining wellbeing (Athota et al., 2019; Creed & Evans, 2002). Personality variables not only explain differences, they also moderate the effects of other individual and situational determinants of psychological wellbeing.

The third implication is in line with the assumption that the context has a controlling effect (Ryan & Deci, 2002) and our assumption that the moderating effects of locus of control differ among the three groups was partly supported. Our results revealed that interaction effects of ambiguity intolerance and internal locus of control on eudaimonic wellbeing were substantial among the unemployed and self-employed but not among the salary-employed. On the other hand, the interaction effects of ambiguity intolerance and external locus of control were substantial for all three employment status groups. Particularly, the unemployed and self-employed with a high internal locus of control or low external locus of control tend to maintain high eudaimonic wellbing at all levels of ambiguity intolerance. Given that eudaimonic wellbeing for individuals in unemployment and self-employment with a high internal locus of control or low external locus of control tends to be very high, internal locus of control may have a trumping effect such that other factors are not pertinent in explaining psychological wellbeing when the internal locus of control is high or when the external locus of control is low.

Similarly, our results indicate that psychological capital buffers against the negative effects of ambiguity intolerance on psychological wellbeing. Although we generally found no interaction effects of ambiguity intolerance and psychological capital on eudaimonic wellbeing, the three-way interactions were significant, indicating that the effects of psychological capital on the relationship between ambiguity intolerance and psychological wellbeing are dependent on employment status. The probe of the moderation revealed significant interaction effects for the self-employed and salary-employed but not for the unemployed. Therefore, we cannot confidently claim a buffering effect of psychological capital against the negative effects of ambiguity intolerance on the psychological wellbeing of the unemployed. However, the buffering effect can be claimed for the salary- and self-employed groups. We need to note however that the regression plots indicate that the unemployed with higher psychological capital report higher levels of eudaimonic wellbeing at all levels of ambiguity intolerance than their counterparts with lower levels of psychological capital. The psychological capital theory highlights four important facets of confidence, hope, optimism, and resilience (Luthans et al., 2004; Luthans and Youssef-Morgan 2017) which are essential to maintaining health and vitality. Consequently, it buffers against negative influences on eudaimonic wellbeing among people in business or in complex work situations or statuses, which corroborates previous empirical findings for example among the self-employed (Baron et al., 2016). These resources are important for sustaining a high level of quality of life and wellbeing among populations in difficult circumstances such as unemployed, homeless, and immigrants (Bak-Klimek et al., 2014; Munoz et al., 2016). Hence, the strength of psychological resources is essential for individuals in difficult work situations in maintaining a high level of eudaimonic wellbeing.

### Limitations

This study has several strengths that highlight its contribution to knowledge on the association between psychological attributes and wellbeing among individuals in different employment statuses. Particularly, we use a robust sample comprising of unemployed, salary, and self-employed individuals from two countries, which enhances the generalizability of our findings. However, several limitations should be taken into consideration when applying our findings. First, we used Samman’s (2007) suggested measures for eudaimonic wellbeing. However, this approach is not yet well-validated. Future studies could focus on validating this approach to establish where it is comparable or superior to well-known existing measures. Also, whereas we argue from the viewpoint that each of the three employment statuses involves some unique uncertainties, we did not measure these uncertainties. Rather, we measured the individual disposition to tolerate such ambiguities (ambiguity intolerance). Future-related research therefore could add more value by measuring the unique ambiguities of each employment status that could predict eudaimonic or other forms of wellbeing.

Second, we collected cross-sectional data, which limits making firm conclusions about causal relations among the study variables. Particularly, this is associated with common methods bias with the potential of inflating variances in the findings (Doty & Glick, 1998). However, it has been observed that common methods bias is not a great challenge when using measures with multiple items and with high reliabilities (Fuller et al., 2016) and when using complex analyses (Evans, 1985). Nonetheless, we suggest that future studies could benefit from using longitudinal data where variations in the wellbeing of people in different employment statuses can be observed at different times. Experimental studies incorporating interventions, for example, could be applied to measure whether an increase in psychological capital for these groups would result in improved psychological wellbeing.

Another limitation is that whereas we collected data from two countries, we only used “country” as a control variable. We did not analyze potential cross-country differences. Whereas the two countries are from the same region with similar socio-economic characteristics, our results indicate that there are differences in eudaimonic wellbeing. Therefore, future studies should consider establishing cross-cultural variations, including comparisons among countries from different regions. Involving more countries would be important especially where differences in statutory benefits or support mechanisms for the unemployed, self-employed, and those in bad jobs can be analyzed. Third, we primarily used self-report measures. Consequently, we cannot rule out social desirability bias. Further research using multiple methods of data collection would be beneficial to understanding the contribution of psychological states to maintaining high levels of psychological wellbeing during times of uncertainty. For example, there exists the “other person-rater version” of the psychological questionnaire that could be used alongside the self-rater version to obtain more objective data.

### Theoretical and Practical Implications

Despite the above limitations, we believe that the study makes important contributions to theory and practice in understanding and promoting the eudaimonic wellbeing of individuals in unemployment, precarious salaried-employment, and self-employment. In line with the deprivation theory, individuals in unemployment face many challenges. However, those in salaried employment in some contexts are also confronted with similar uncertainties. Our results suggest that if individuals are high at ambiguity intolerance, their eudaimonic wellbeing may eventually deteriorate. First, our findings extend the locus of control (and generally personality) theory that it not only predicts eudaimonic wellbeing but also moderates the relationship between other psychological attributes (e.g., ambiguity intolerance) and psychological wellbeing. Secondly, our findings extend positive psychology theory (e.g., Luthans & Youssef, 2007; Rand & Cheavens, 2012; Seligman, 2002) by showing that those with higher psychological resources can maintain higher levels of eudaimonic wellbeing despite intolerance to ambiguities that may be faced by the different employment status groups.

Paying attention to the eudaimonic wellbeing of people in different employment status groups is essential. For the unemployed and salary-employed, it may be essential for obtaining jobs (or better ones) given its implications for their perceived employability (Whelan et al., 2018) and consequently job search behaviors. It also affects the physical health of individuals, families, and performance at work (Ryan, 2009; Steptoe et al., 2015; Wright & Cropanzano, 2000). For those in self-employment, psychological wellbeing is critical for their persistence in their work (Baluku, 2017; Patel & Thatcher, 2014). It is, therefore, necessary that policymakers keep a keen interest in the psychological wellbeing of people in different employment statuses. Our results add to extant findings on the dangers of being unemployed or being employed in a precarious job. The exacerbated existence of unemployment and precarious work is against the global goals of decent work and health for everyone. However, our results suggest that governments and their development partners should not only focus on creating jobs but the quality of jobs matters. Moreover, in addition to skill enhancement programs, interventions to improve psychological wellbeing through enhancement of psychological resources are needed for those who are not yet employed or having jobs they perceive as bad, given that the application of positive psychological resources enhances wellbeing (Baumann & Eiroa-Orosa, 2016).

## Conclusion

In summary, the study revealed that ambiguity intolerance has substantial negative effects on eudaimonic wellbeing among the unemployed, salary-employed, and self-employed. However, we have demonstrated that two psychological resources including internal locus of control and psychological capital are essential to maintaining high levels of eudaimonic wellbeing. They not only predict high psychological wellbeing among these groups, but they also buffer against the negative effects of ambiguity intolerance.

## Data Availability

Data used for this study can be obtained from the corresponding author upon a written request.

## Declarations

This paper has been developed from the larger research project for Dr. Martin Baluku’s Ph.D. dissertation. The project was given ethical clearance by the Philipps-University, Faculty of Psychology Ethics Commission – AZ: 2015-21K. The study was also conducted in conjunction with Makerere University School of Psychology and Kisii University. Clearances were obtained from the Dean and Vince Chancellor for Research in these institutions respectively.
